# Research Progress of Passively Mode-Locked Fiber Lasers Based on Saturable Absorbers

**DOI:** 10.3390/nano15231819

**Published:** 2025-12-01

**Authors:** Jiayi Xie, Tengfei Liu, Xilong Liu, Fang Wang, Weiwei Liu

**Affiliations:** Tianjin Key Laboratory of Micro-Scale Optical Information Science and Technology, Institute of Modern Optics, Nankai University, Tianjin 300350, China; 18233169508@163.com (J.X.);

**Keywords:** artificial SA, real SA, fiber laser, mode-locked

## Abstract

Ultrashort fiber lasers are one of the current research hotspots in the field of lasers. They have the advantages of compact structure and high beam quality. Passively mode-locking using saturable absorbers (SAs) is an important scheme for generating picosecond and femtosecond pulses. A deep understanding of the passive mode-locking mechanism is key to maturing ultrafast laser technology. In recent years, the passively mode-locking technology of SAs has been improved in material systems, device preparation, and cavity structures. SAs are primarily divided into artificial SAs and real SAs. Real SAs primarily include semiconductor saturable absorption mirrors (SEASAMs) and nanomaterials. Artificial SAs primarily include nonlinear optical loop mirrors (NOLMs), nonlinear multimode interference (NLMMI), nonlinear polarization rotation (NPR), and the Mamyshev oscillator. Herein, we mainly review passively mode-locked fiber lasers employing various SAs, as well as their working principles and technical characteristics. By focusing on the representative achievements, the developmental achievements of ultrafast lasers based on SAs are demonstrated. Finally, the prevailing challenges and promising future research directions in SA’s mode-locking technology are discussed.

## 1. Introduction

Ultrashort laser pulse generation represents a revolutionary technology that has transformed numerous fields, particularly in ultra-fine processing [1], time-resolved spectroscopy [2], high-density information storage [3], telecommunications [4], and strong field physics [5]. It centers on the emission of extremely short-duration laser pulses, ranging from picosecond (ps) to femtosecond (fs). The primary methods for obtaining ultrashort pulse lasers include Q-switching [6], active mode-locking [7], passively mode-locking [8], and synchronous pumping [9]. Among these, passively mode-locking offers distinct advantages such as a simple structure, the elimination of external modulators, and ease of full-fiber integration. It is considered one of the most effective and practical approaches for generating ultrashort pulses. As a prominent representative of third-generation laser technology, fiber lasers possess inherent advantages over traditional solid-state and gas lasers, including high conversion efficiency, excellent beam quality, effective thermal management, structural compactness, exceptional stability, and ease of integration. Consequently, the integration of passively mode-locking with fiber lasers to create passively mode-locked fiber lasers has become a quintessential approach for achieving cost-effective and high-performance ultrashort pulse sources.

The core physical mechanism of passively mode-locking relies on nonlinear effects within the laser cavity, enabling the self-shaping, self-narrowing, and self-initiation of optical pulses. This process is based on the saturable absorption effect, a concept first proposed in the 1960s [10], where nonlinear elements selectively absorb low-intensity light within the cavity. Dyes were the first SAs discovered, T. H. Maiman introduced dye into the ruby laser. At low intensities, the dye SA absorbs light, placing the laser in a loss state, preventing emissions. As light intensity further increases, the dye’s absorption capacity diminishes, and the dye SA reaches saturation, resulting in laser outputs at high-intensity light pulses. Due to their superior physical and chemical stability compared to dyes, crystals gradually replaced dyes as the preferred SAs in subsequent years. In 1996, Keller et al. introduced the semiconductor saturable absorber mirror (SESAM) into solid-state lasers [11], enabling passively mode-locking and marking a significant advance in the field. SESAMs exhibit high environmental stability and a high damage threshold. However, they suffers from the drawback of high manufacturing costs. After years of efforts by scientists, nanomaterials such as carbon nanotubes (CNTs) [12], graphene [13], black phosphorus (BP) [14], topological insulators (TIs) [15], transition metal dichalcogenides (TMDs) [16], and other nanomaterials [17,18,19,20] have been found to possess excellent properties, demonstrating strong potential as SAs in fiber laser systems. These materials are categorized as real SAs, yet their practical application is constrained by fixed saturable absorption properties and reaction times. In the 1990s, several artificial SAs were created for mode-locking, including nonlinear optical loop mirrors (NOLMs) [21], nonlinear multimode interference (NLMMI) [22], nonlinear polarization rotation (NPR) [23], and the Mamyshev oscillator [24]. The operation of a NOLM can be described using the model of a figure-eight cavity. Two counter-propagating beams of varying intensities pass through the loop, causing intensity-dependent nonlinear phase shifts that compress the pulses and induce mode-locking. In contrast, NLMMI utilizes the superposition of multiple modes in a multimode fiber. The intensity-dependent refractive index affects the length of the transmission beat. Pulse narrowing and mode-locking can be accomplished by accurately adjusting the length of a multimode fiber. NPR generates ultrashort pulses by regulating the optical pulse’s polarization state and making use of the dependence of nonlinear optical effects on the input pulse’s intensity, using a polarizer to select the appropriate polarization. The Mamyshev oscillator was designed to increase the peak output of fiber lasers. After spectrum broadening with self-phase modulation (SPM) in high-power pulses, spectral filtering is used to reduce low-power components, resulting in pulse narrowing. Artificial SAs have a large operational bandwidth but high structural complexity.

**Figure 1:**
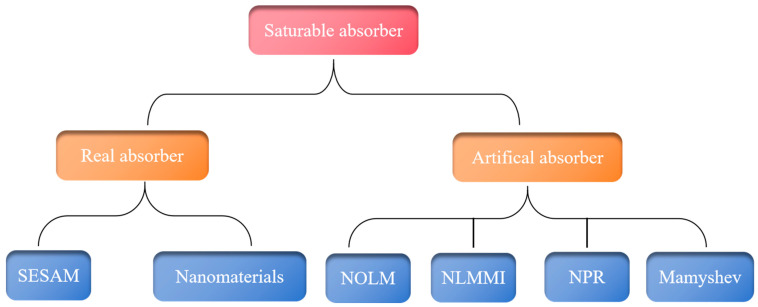
Classification diagram of SAs.

Figure 1 shows a categorization diagram of SAs. Key parameters of SAs encompass the wavelength response range, saturable intensity, relaxation time, and modulation depth. The precise selection of SA parameters is crucial for enabling ultrashort fiber lasers with self-starting capabilities, robust environmental stability, and tunable pulse parameters. To expand our understanding of ultrashort fiber lasers, this paper examines current advances in several types of passively mode-locked fiber lasers with SAs, including novel mode-locking approaches developed in recent years. Their technological strengths and the current obstacles are explained. Furthermore, the application potential and development trends of ultrashort fiber lasers are investigated.

## 2. Characteristics of SAs

Modulation depth (Δ*T*): Δ*T*, defined as the ability of a saturable absorber to modulate light intensity, is quantified by the maximum change in the absorption or reflectance. The modulation depth can be expressed by the following equation, where *q*_0_ is the absorption amplitude loss coefficient:

(1) $$\Delta T=1-2e^{-2q_{0}}$$

The modulation capability of the SA depends on its optical absorption characteristics, thickness, and the structure of the saturable absorber device. A low modulation depth results in comparable loss for both the edges and the center of the pulse, such that it prevents mode-locking from self-starting. A large modulation depth facilitates strong pulse shaping in SAs, enabling the generation of short pulse widths and enhancing the self-starting capability. However, it may lead to the unstable Q-switched mode-locking.

Non saturable loss is characterized by a linear increase in the absorption of SAs with increasing photon density at low levels. Scattering and absorption are the primary sources of non-saturable loss. Low non-saturable loss is helpful for the laser to achieve high energy-conversion efficiency. However, excessive non-saturation loss can lead to the accumulation of thermal effects, which not only degrades the laser’s performance but also damages the optical components.

Saturated fluence (*F_sat_*) refers to the luminous fluence at which the transmittance of an SA reaches saturation. It can be defined by the following equation, where σ*_A_* is the absorption cross section and *hν* is the photon energy:

(2) $$F_{sat}=\frac{h\nu }{2\sigma_{A}}$$

In mode-locking applications, the incident pulse fluence needs to be several times the saturation fluence to achieve continuous mode-locking. If the saturation fluence is excessively high, the pulse fluence required for continuous mode-locking will also be higher, which can lead to optical damage. Conversely, if it is too low, the SA becomes over saturated, making the laser prone to generating multiple pulses at low power levels.

## 3. Real SA

This section describes and discusses two primary types of real SAs used for mode-locking in fiber lasers, including SESAMs and nanomaterials. Their properties, together with the novel contributions demonstrated by researchers, are examined.

### 3.1. Semiconductor Saturable Absorber Mirror

Owing to the superior properties of semiconductor materials, the SESAM enables standalone mode-locking within the cavity, delivering powerful saturable absorption and operational stability. Modulation depth, operating wavelength, and recovery time can all be freely adjusted during SESAM manufacture. It is a commonly used SA in passively mode-locked fiber lasers because of its simple integration onto fiber mirrors, which permits an all-fiber architecture.

The semiconductor material composition of the SESAM should be adjusted accordingly for fiber lasers with varying center wavelengths [25]. For mode-locked Yb-doped and Nd-doped fiber lasers [26], the SESAM structure typically consists of an InGaAs quantum well and a GaAs/AlAs Bragg reflector. For mode-locked Bi-doped and Nd-doped fiber lasers [27], the SESAM structure typically consists of a GaInNAs quantum well and a GaAs/AlAs Bragg reflector. For Er-doped and Tm-doped fiber lasers with a center wavelength exceeding 1.5 μm [28], the SESAM structure typically consists of an InGaAs/InP quantum well and a InGaAs Bragg reflector. For Er-doped and Tm-doped fiber lasers with a center wavelength exceeding 2 μm [29], the SESAM structure typically consists of an GaInSb quantum well and a AlAsSb/GaSb Bragg reflector.

**Figure 2:**
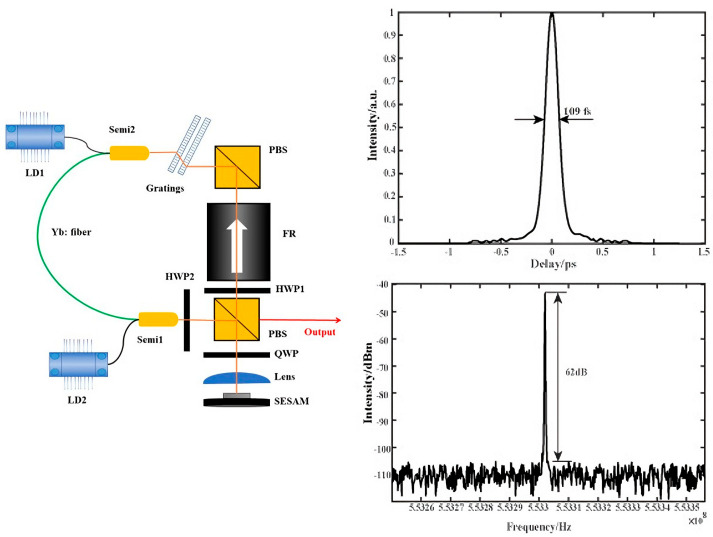
SESAM mode-locked polarization-maintaining fiber laser with dispersion compensation using integrated grating pairs and the test results of pulse width and repetition rate (Semi, semi-wavelength-division multiplexer; PBS, polarization beam splitter; HWP, half-wave plate; QWP, quarter-wave plate; FR, Faraday rotator) [34].

An Nd-doped fiber laser with anti-resonant SESAM mode-locking was shown by Ober et al. in 1993 [30]. In their setup, pulses as short as 260 fs could be generated. However, the cavity required an intra-cavity lens and a group delay dispersion line, making the design very complicated. Zhang et al. [31] demonstrated a linear-cavity SESAM mode-locked fiber laser constructed from a polarization-maintaining fiber (PMF). By using the versatility of a PMF loop mirror, they simplified the system’s design, producing a dissipative soliton pulse at a 2.1 nJ pulse energy with pulse width of 24 ps. According to Okhotnikov et al. [32], a near-resonant SESAM is especially well suited for Yb-doped fiber lasers because it can accomplish stable mode-locking in a short fiber cavity. Zhang et al. [33] introduced high-gain Yb-doped photonic crystal fiber into a mode-locked fiber laser, employing a 0° angle fiber end face as the cavity mirror. The filtering effects of the SESAM and two gratings were used to produce stable mode-locked functioning. Continuous tunability from broadband-filtering mode-locking to narrowband-filtering mode-locking is accomplished by varying the filtering degree. As shown in Figure 2, Yu et al. [34] proposed an SESAM mode-locked polarization-maintaining Yb-doped fiber laser, incorporating an integrated grating pair for dispersion compensation. This setup produced an ultrashort pulse with a 109 fs pulse width and 553 MHz repetition rate for the first time under similar circumstances. Moreover, a number of commercial SESAM mode-locked fiber lasers have been reported. Tian et al. [35] used an all-normal dispersion cavity design to produce a high-energy laser pulse output of 4.3 nJ. A narrow bandwidth SESAM was used by Liu et al. to establish stable mode-locking of a Yb-doped fiber laser, allowing for the creation of high-repetition-rate pulses [36]. Hekmat et al. [37] reduced the pulse duration in an SESAM mode-locked fiber laser by implementing a Faraday rotating mirror, thereby enabling ultrashort pulse generation. Table 1 summarizes some of the achievements in fiber laser mode-locking using an SESAM as an SA. SESAM fabrication relies on costly molecular beam epitaxy (MBE) systems, which are challenging to implement in low-cost and popular fiber lasers.

### 3.2. Nanomaterials

This section describes and discusses five primary types of nanomaterial SAs used for mode-locking in fiber lasers, including CNTS, graphene, BP, TIs, and TMDs. A brief comparison of various nanomaterials can be found in Table 2.

#### 3.2.1. Carbon Nanotubes

**Figure 3:**
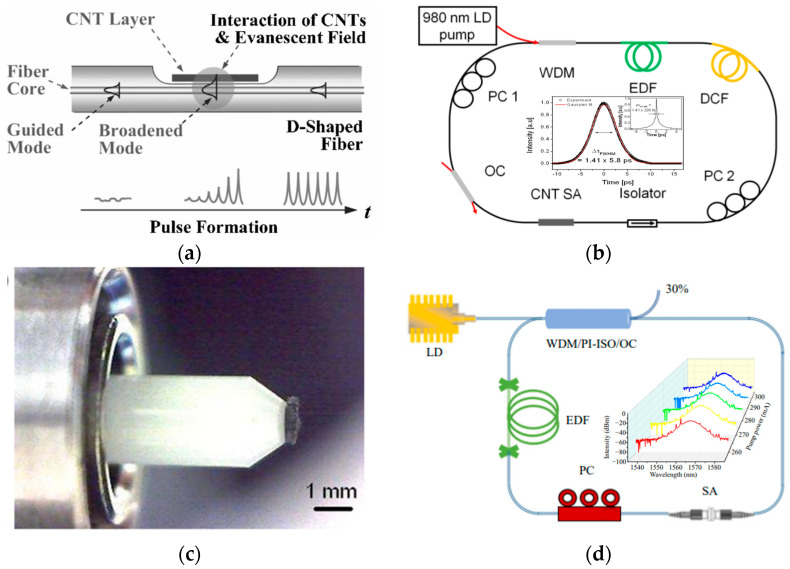
Using CNTs for mode-locking in fiber lasers: (**a**) interactions between CNTs and transient fields [61]; (**b**) fiber laser composed of positive-dispersion ring cavity and the pulse width (WDM, wavelength division multiplexing coupler; EDF, Er-doped fiber; DCF, dispersion-compensating fiber; PC, polarization controller; OC, output coupler) [64]; (**c**) the end face of fibers coated with CNTs [65]; (**d**) passively mode-locked C-band fiber laser employing carbon nanotubes as saturable absorbers (PI-ISO, polarization-independent isolator) [66].

Carbon nanotubes (CNTs) are tubular nanostructures composed of sp^2^-hybridized carbon atoms. They were first discovered in 1991 by Sumio Iijima at NEC’s Fundamental Physics Laboratory in Japan [59]. CNTs are a kind of one-dimensional nanomaterial, distinguished by radial dimensions that are limited to the nanoscale. In the axial direction, CNTs can reach sizes of hundreds of microns. Single-walled CNTs (SWCNTs) and multi-walled CNTs (MWCNTs) are separated based on the quantity of graphite sheets that make up the CNT. Two or more SWCNTs can form an MWCNT, with a spacing of 1 nm between them. In 2003, Set et al. [60] introduced SWCNTs into the ring cavity and linear cavity of a fiber laser, achieving 1 ps mode-locked laser pulse output at a wavelength of 1550 nm. As illustrated in Figure 3a, interactions between CNTs and transient fields lead to mode-locking [61]. Song et al. [62] deposited CNTs onto the side of a tapered fiber in an Er-doped fiber laser, gaining pulses with a pulse width of 829 fs and a spectral width of 3.7 nm. Martinez et al. [63] injected a CNTs solution into the self-made slit. The laser produced ultrashort pulses with varying pulse widths and wavelengths when the slit length varied. As shown in Figure 3b, Im et al. [64] deposited CNTs onto a side-polished fiber and applied it in a positive-dispersion ring cavity, yielding a mode-locked pulse with an initial width of 5.8 ps. Another popular method of mode-locking involves CNTs interacting directly with the optical electric field. As seen in Figure 3c, the fiber’s end face is coated with CNTs [65]. Luo et al. [66] prepared the CNT SA. As illustrated in Figure 3d, by fastening CNT SAs between two fiber end faces using an adapter, they achieved mode-locking of a C-band fiber laser. However, CNTs possess a large surface area and exhibit higher unsaturated losses, which limits the application range of CNTs.

#### 3.2.2. Graphene

**Figure 4:**
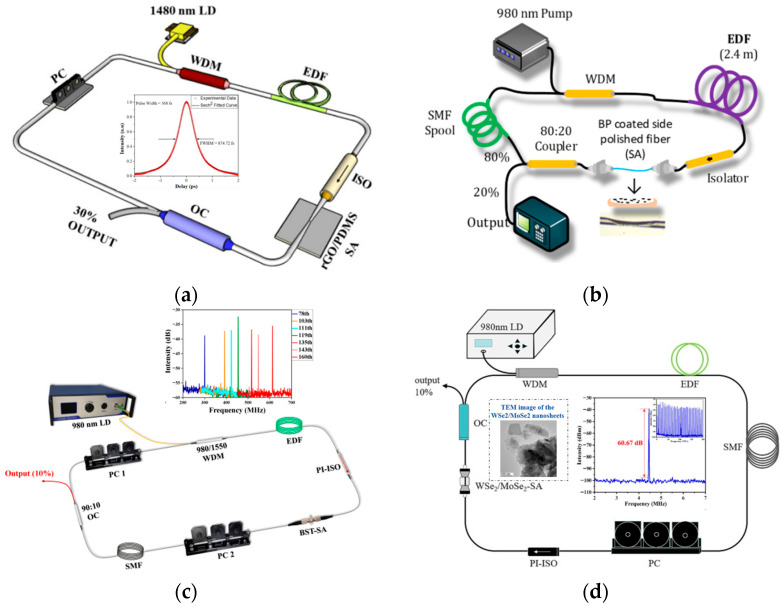
Using graphene, BP, Tis, and TMDs for mode-locking in fiber lasers: (**a**) L-band femtosecond fiber laser based on graphene SA [73] (rGO/PDMS, graphene oxide/polydimethylsiloxane); (**b**) mode-locked fiber laser with BP-coated D-shaped fiber [74]; (**c**) fiber laser based on Bi_2_SeTe_2_ SA mode-locking [75] (BST, Bi2Se2Te); (**d**) WSe_2_/MoSe_2_ heterojunction mode-locked fiber laser, electron microscope image of WSe_2_/MoSe_2_-SA [76].

Graphene is composed of sp^2^ hybrid carbon atoms, which is a single layer planar material that resembles a honeycomb. Graphene is a two-dimensional quantum system that exists stably at room temperature, and it is also the basic unit of CNTs. In 2004, Novoselov et al. [67] exfoliated graphene from graphite flakes. As a zero-bandgap material, it exhibits unique light-saturated absorption properties. In 2009, Bao et al. [68] first implemented stable mode-locking with the pulse width of 756 fs, using graphene as an SA. Graphene at the atomic layer exhibits wavelength-insensitive ultrashort saturation absorption, making it suitable as a full-bandwidth mode-locking component. Lau et al. [69] transferred graphene nanoplate powder onto the end face of a fiber, generating mode-locked pulses with a central wavelength of 1558.35 nm. Wang et al. [70] employed graphene for mode-locking in a Tm-doped fiber laser, delivering mode-locked pulses with a center wavelength of 1953.3 nm. Zhang et al. [71] proved through experiments that graphene with atomic-layer thickness can be used as an ideal mode-locked device. They adjusted the pump power and polarization controller to generate wavelength-tunable dissipative solitons. Graphene derivatives have also been used in mode-locked fiber lasers. Lin et al. [72] obtained switchable single-wavelength and dual-wavelength femtosecond solitons in Er-doped fiber lasers using carboxylated graphene oxide. Ng et al. [73] fabricated reduced graphene oxide/polydimethylsiloxane (PDMS) as an SA. As illustrated in Figure 4a, they achieved 568 fs ultrashort pulse output in the L-band. The low electronic density of states near the Dirac point of graphene limits the number of electronic states available for optical absorption, thereby resulting in a low modulation depth for graphene SAs.

#### 3.2.3. Black Phosphorus

Black phosphorus (BP) has several advantages, such as the layer-dependent bandgap, high carrier mobility, and a wide spectral response range. Its bandgap can be tuned over a wide range, making it suitable for use as a broadband SA in fiber lasers. In 2015, Chen et al. [77] used BP SAs to realize a Q-switched and mode-locked pulse in an Er-doped fiber laser. The same year, Sotor et al. [78] demonstrated ultrashort pulse generation in a Tm-doped fiber laser, where BP served as the SA. The laser delivered output pulses with a central wavelength of 1910 nm and a pulse duration of 739 fs. Zhang et al. [79] prepared BP dispersions of various sizes using liquid-phase exfoliation, then measured their nonlinear absorption properties via Z-scan spectroscopy. The results indicate that the saturable absorption behavior of BP is almost independent of particle size in the visible spectrum, but decreases with smaller particles in the near-infrared region. Subsequently, Qin et al. [80] fabricated a mid-infrared BP SA by transferring mechanically exfoliated BP onto a gold-coated mirror, demonstrating the feasibility of its mode-locking in mid-infrared fiber laser. To further extend the laser’s wavelength range, Qin et al. [81] employed the same method to fabricate a BP SA. By employing dual-wavelength pumping from a 970 nm LD and a 1973 nm Tm-doped fiber laser in the Er:ZBLAN gain medium, they achieved mode-locked pulses at 3489 nm. However, the pulse width of the laser output was not measured. To enhance the damage threshold (>0.0031 mJ/cm^2^ [77]) of the material and avoid large insertion loss caused by trapping BP within the fiber joint, as seen in Figure 4b, Alghamdi et al. [74] recently coated BP onto side-polished D-shaped fibers. This approach enabled the generation of mode-locked pulses with a pulse width of 1.17 ps. Nevertheless, the isolated BP readily reacts with air and water, leading to oxidation. This poses a threat to the long-term stable operation of fiber lasers that utilize BP as an SA. Several strategies have been proposed to improve the environmental stability of BP. These include physical isolation using protective coatings of aluminum oxide or hexagonal boron nitride, as well as chemical modification through surface treatment with phenyl viologen or ionic liquids [82].

#### 3.2.4. Topological Insulators

Topological insulators (TIs) are a new type of quantum material. Their interior exhibit an insulating state, while their surface exhibit a metallic state. TIs have been extensively studied in condensed matter physics. Compared to graphene, TIs exhibit high modulation depth (9.8% [83]), high damage threshold (1.354 mJ/cm^2^ [84]), and broadband saturable absorption. In 2012, Bernard et al. [85] initially investigated the saturated absorption properties of Bi_2_Te_3_ at the wavelength of 1550 nm, and predicted that ultrashort pulse mode-locking could be realized by using TIs. The same year, Zhao et al. [86] achieved mode-locking in an Er-doped fiber laser using a Bi_2_Te_3_ SA. The modulation depth of Bi_2_Te_3_ reached 95.3%, with an output pulse width of 1.86 ps. Dou et al. [75] fabricated the Bi_2_Te_3_ SA using the thermal precipitation method, attaining mode-locked pulses with a pulse width of 46 ps in the multi-pulse region. Furthermore, numerous studies have demonstrated that other TIs can also function as innovative SAs for mode-locking. By inserting Sb_2_Te_3_ SA into a side-polished fiber, Sotor et al. [87] produced ultrashort pulses with a center wavelength of 1561 nm and a pulse width of 270 fs. Haris et al. [88] fabricated Bi_2_Se_3_ SAs via optical deposition and integrated them into the Er-doped fiber laser, generating single and bound solitons at different cavity lengths. Liu et al. [89] used liquid-phase exfoliation to create a Bi_2_SeTe_2_ SA. With a pump power of 89.9 mW, they were able to accomplish fundamental mode-locking. As shown in Figure 4c, by increasing the pump power, they realized harmonic mode-locking from the 79th to the 160th order. Jone et al. [83] calculated the absorption spectra of several TIs using density functional theory, demonstrating that Bi_2_Se_3_ offers greater merits than Bi_2_Te_3_ for mode-locking mid-infrared fiber lasers. However, thin film TI materials cannot replace traditional ultrashort optical devices. Current fabrication methods for TIs face challenges with thermal and mechanical stability, along with limited sample reproducibility. This means that TIs might further increase their application potential with the improvement of processing technology.

#### 3.2.5. Transition Metal Dichalcogenides

Transition metal dichalcogenides (TMDs) are substances that are commonly denoted by the formula MX_2_, in which X is a chalcogen element and M is a transition metal. The energy band structure of TMDs can be altered by varying the number of layers, which allows for control over optical response and carrier mobility. The output pulses from mode-locking lasers that use TMDs as SAs show a high degree of flexibility. TMDs display substantial third-order polarizability under intense optical excitation. Additionally, single-layer TMDs materials exhibit an optical absorption of approximately 10% at resonance wavelengths, which is greater than graphene’s absorption rate of 2.3%. This suggests that high-performance saturable absorption can be attained by TMDs.

Wang et al. [90] investigated the saturable absorption properties of MoS_2_ films. They showed that under identical conditions, MoS_2_ exhibits better saturable absorption performance than graphene in the near-infrared region. In 2014, Zhang et al. [91] used MoS_2_ as an SA to realize dissipative soliton mode-locking in a Yb-doped fiber laser, and the output pulse width was 800 ps (during the mode-locking of dissipative solitons, normal dispersion occurs within the cavity, resulting in a broad pulse width). Then, Zhang et al. [92] used MoS_2_ polymer film to mode-lock an Er-doped fiber laser and produced a picosecond pulse laser with a wavelength tuning range of 1535 nm to1565 nm. Subsequently, some experiments employing TMDs such as WS_2_ [93], MoSe_2_ [94], WSe_2_ [95], PdSe_2_ [96], and VSe_2_ [76] in passively mode-locked fiber lasers have been reported. With the improvement of material preparation technology, researchers found that doping TMDs or constructing heterojunctions can enhance the energy band structure of TMDs. Du et al. [97] constructed the graphene/WS_2_ heterojunction SA by layer transfer of monolayer graphene and WS_2_. By means of interlayer charge transfer and energy band alignment, the saturation intensity was halved, and the modulation depth was increased by 4.8 times in comparison to pure WS_2_. Recently, as shown in Figure 4d, Pang et al. [98] employed a self-fabricated WSe_2_/MoSe_2_ heterojunction SA in a Er-doped fiber laser, generating bright-dark soliton pairs with a repetition rate of 4.459 MHz. The dimensions of TMDs are constrained by the fiber core diameter. In order to avoid material damage (>0.0646 mJ/cm^2^ [99]), the power of the fiber laser should not be too high. It is encouraging that increasing the length of the doped fiber can enhance gain, thereby compensating for material loss, making it easier to achieve mode-locking in fiber lasers.

Some of the advancements in fiber laser mode-locking with nanomaterial SAs are compiled in Table 3.

## 4. Artificial SA

This section describes and discusses four types of artificial SAs used for mode-locking in fiber lasers, including nonlinear optical loop mirrors (NOLMs), nonlinear multimode interference (NLMMI), nonlinear polarization rotation (NPR), and the Mamyshev oscillator. The working principle and technical advantages are explained here, and the innovative contributions made by researchers are also examined.

### 4.1. Nonlinear Optical Loop Mirror

**Figure 5:**
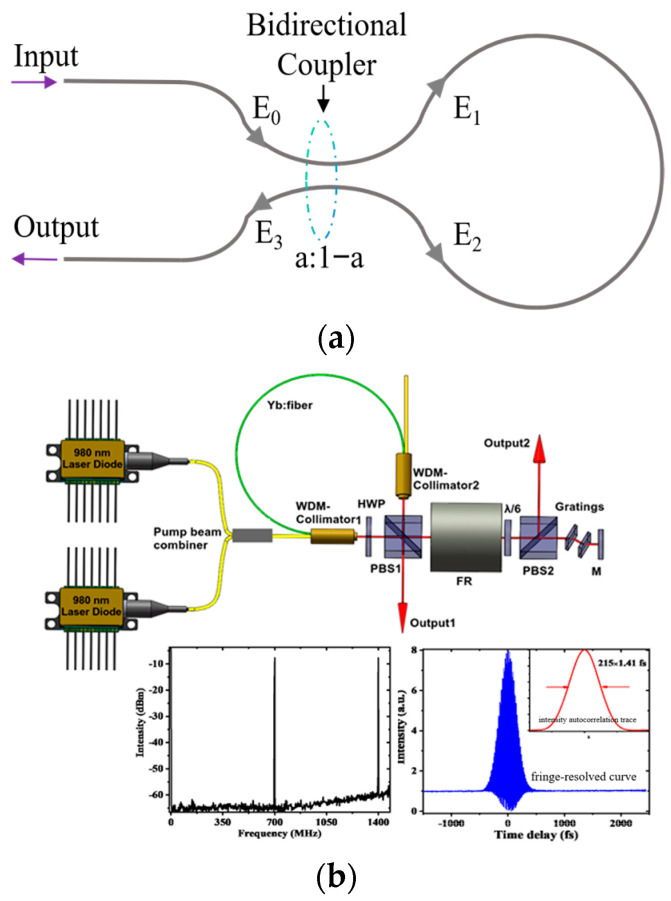
Using NOLM for mode-locking in fiber lasers: (**a**) nonlinear fiber ring mirror device; (**b**) 700 MHz mode-locked Yb–fiber laser with a biased NALM and the text results of repetition rate and pulse width [104].

Nonlinear optical loop mirrors (NOLMs) can be described using the model shown in Figure 5a. Due to its shape resembling the number “8”, it is known as the figure-eight cavity [102]. A 2 × 2 coupler with a splitting ratio of a:1-a forms the basis of the NOLM. The coupler splits the incident light E_0_ into two counter-propagating beams with varying optical field strengths. When the two beams of light return through the loop, they accrue distinct nonlinear phase shifts due to nonlinear processes inside the cavity. At this time, the NOLM functions as a fast SA, resulting in high transmittance for the high-intensity portion of the pulse and low transmittance for the low-intensity portion of the pulse. Pulse narrowing and stable mode-locked operation are ultimately achieved. When the coupling splitter’s splitting ratio differs, causing two optical pulses to undergo varying nonlinear phase shifts, the device is termed an NOLM. When the amplification effect of the gain fiber differs between two optical pulses, causing different nonlinear phase shifts in the pulses, the device is termed a nonlinear optical amplifier (NALM). In contrast to material-based SAs, the NOLM has superior damage tolerance and faster response times. Since the Naval Research Laboratory in Washington DC reported the first stable traditional soliton pulse mode-locked fiber laser based on NOLMs in 1991 [103], scholars have conducted more detailed research on NOLMs.

To enhance the stability of fiber lasers with NOLMs for mode-locking, Aguergaray et al. proposed a fully polarization-maintaining figure-eight cavity mode-locked fiber laser. Its primary technology uses PMF in place of single-mode fiber (SMF). By adjusting the fiber length and coupling ratio in the NOLM, Aguergaray et al. [105] achieved mode-locked pulse with a central wavelength of 1027 nm. This laser showed stable mode-locking maintenance during almost 3000 h of continuous operation. To improve pulse controllability, Zhou et al. [106] used a dual-gain structure in a figure-eight cavity. The pulse output energy was 32 nJ, with a peak-to-peak power ripple of 0.9% over 24 h. Nevertheless, structural complexity is a problem with this solution. To achieve sufficient nonlinear phase shift for two beams of light within a figure-eight cavity structure, it is necessary to incorporate a longer length of SMF. This leads to a lower repetition rate in fiber lasers, thereby excluding NOLMs from high-repetition-rate applications. Therefore, researchers designed a “9” character cavity structure. The shorter cavity length of the nine-character cavity allows for higher laser repetition rates in design, but this leads to insufficient accumulation of nonlinear phase shift, making it difficult for the laser to achieve self-starting mode-locking. Zhou et al. [107] constructed a non-polarization-maintaining (NPM) nine-character cavity fiber laser. By placing a Faraday rotator between two quarter wave plates to achieve the initial phase shift, they obtained pulses with a repetition rate of 80 MHz. This resulted in a more compact nine-cavity fiber laser, as shown in Figure 5b. Liu et al. [104] adopted a solution combining polarization-maintaining gain fiber with spatial components. At a pump power of 710 mW, the mode-locked pulses exhibited a repetition rate of 700 MHz and a pulse width of 215 fs.

Due to the low gain of traditional rare-earth ions in the visible light spectrum, the development of visible light fiber lasers has been slow. Compared to other mode-locking mechanisms in fiber lasers, NOLMs are not only wavelength-insensitive but also tolerate significant cavity losses. To leverage this advantage and broaden the wavelength coverage, fluoride-based fiber lasers with figure-eight cavities have emerged. This approach also necessitates high-energy blue semiconductor lasers as the pumps. In 2020, Zou et al. [108] realized the first visible mode-locking light fiber laser using the NOLM. They controlled the cavity polarization by designing a pattern adapter and connecting Pr^3+^/Yb^3+^ co-doped ZBLAN fiber with 460 HP single-mode fiber, achieving a dissipative soliton with a central wavelength of 635 nm and a pulse width of 567 ps. Recently, Zou et al. [109] pumped a double-clad Pr^3+^:ZBLAN fiber using a multimode diode laser and employed an all-fiber NOLM for mode-locking. They first realized a crimson fiber laser with a central wavelength of 717.5 nm.

### 4.2. Nonlinear Multimode Interference

**Figure 6:**
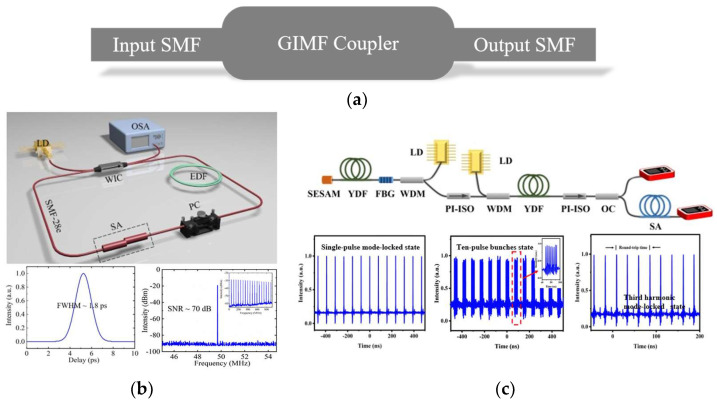
Using NLMMI for mode-locking in fiber lasers: (**a**) NLMMI device; (**b**) mode-locked fiber laser based on misaligned GIMF and the text result of pulse width and repetition rate [111] (WIC, waveguide isolator coupler; OSA, optical spectrum analyzer); (**c**) mode-locked fiber laser with NPR and NLMMI and the text result of pulse sequence, ten-pulse bunches, and third harmonic mode-locked sequence [112] (FBG, fiber Bragg grating; YDF, Ytterbium-doped fiber).

As shown in Figure 6a, NLMMI usually consists of SMF and multimode fiber (MMF). MMF has the advantages of high damage threshold (3.68 J/cm^2^ [110]), stable performance, and low price, which attracts scholars to study its saturable absorption characteristics. NLMMI effects refer to the phenomenon where the fundamental mode coupled from an SMF into an MMF excites higher-order modes. Nonlinear mode loss happens when these higher-order modes are coupled back into the SMF from the MMF. Graded-index MMF (GIMF), which is commonly used in NLMMI, propagates all guided modes at particular wavelengths at almost comparable group velocities. Self-phase modulation (SPM) and cross-phase modulation (XPM) in the nonlinear medium cause refractive index dependency on optical power, which affects the transmission beat length. The degree of the saturable absorption effect can be controlled by adjusting the fiber length. On the one hand, low power signals will be divergent in MMF because of the mismatch in core diameter. In contrast, high power signals will continue to be transmitted in MMF because of the self-focusing effect.

In 2013, Nazemosadat et al. [113] proposed the SMF-GIMF-SMF structure and analyzed its feasibility as a nonlinear switch and SA. Jung et al. [114] fabricated an NLMMI SA using a hollow core fiber coated with bismuth telluride. This SA also functions as a band-pass filter. They attained pulses with a pulse width of 46 ps. In order to improve the signal-to-noise ratio (SNR), as can be seen in Figure 6b, Dong et al. [111] dislocated a 3 cm GIMF and a 7 cm GIMF to form a SMF-GIMF-SMF structure, and combined it with NPR to realize mixed mode-locking. By adopting two different mode-locking mechanisms, the laser can output shorter pulses. The hybrid mode-locked fiber laser produces pulses with a pulse width of 1.8 ps and an SNR of 70 dB. Additionally, it can perform mode-locking from the fundamental frequency to the 10th harmonic. Subsequently, an all-fiber mode-locked laser that can switch between single-wavelength and dual-wavelength operation was described by Lin et al. [115]. This was achieved by configuring an NLMMI using the tapered SMF and the tapered GIMF. The switching between single wavelength and dual wavelength is completed by adjusting the loss in the cavity and the polarization controller (PC). As illustrated in Figure 6c, recently, Duan et al. [112] built upon the research of Dong and Lin to establish an NLMMI-NPR mode-locking mechanism in a Yb-doped fiber line cavity, achieving various pulse states including single pulse mode-locking, multi pulse beams, and harmonic mode-locking. Compared to other mode-locking techniques, NLMMI provides full fiber integration, controllable modulation depth, and wavelength tunability. Fiber lasers based on NLMMI mode-locking hold great promise for the development of higher power and more compact lasers.

### 4.3. Nonlinear Polarization Rotation

**Figure 7:**
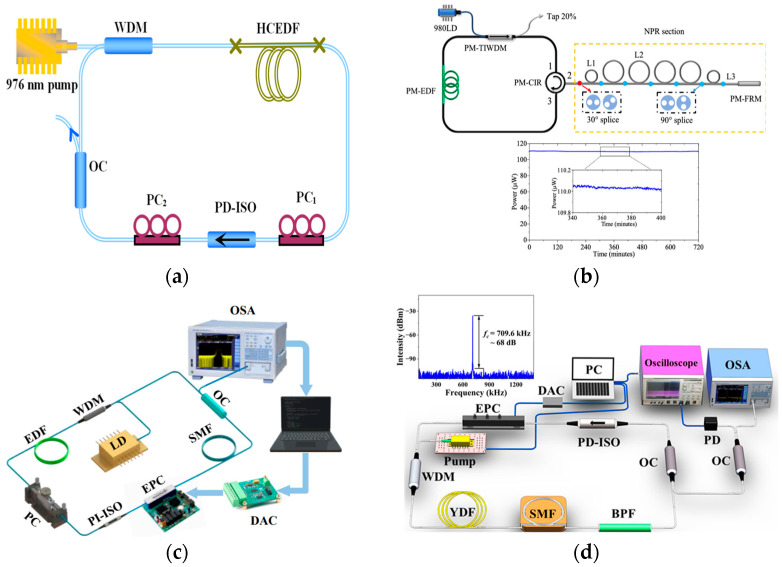
Using NPR for mode-locking in fiber lasers: (**a**) NPR device (HCEDF, high-concentration erbium-doped fiber) [116]; (**b**) L-band fully polarization-maintaining fiber laser with high stability (PM-TTWDM, PM tap-isolating wavelength-division multiplexing; PM-FRM, PM Faraday rotation mirror; PM-CIR, PM circulator) [121]; (**c**) intelligent mode-locking device (EPC, electric polarization controller; DAC, digital-to-analog converter) [122]; (**d**) intelligent mode-locked fiber laser based on particle swarm optimization. (The particle swarm algorithm is an intelligent algorithm that simulates the cooperative foraging behavior of bird flocks. By searching for optimal cavity configurations and operating conditions, it rapidly achieves laser output with target performance. This optimization algorithm introduces fitness function to evaluate the running state of laser. PD-ISO, polarization-dependent isolator; BPF, band-pass filter) [123].

NPR produces ultrashort pulses by manipulating the polarization state of the pulse and extracting a specific polarization using a polarizer. Figure 7a shows a schematic diagram of a fiber laser based on NPR mode-locking [116]. The polarization-dependent isolator (PD-ISO) functions as both an isolator and a polarizer. Light passing through the PD-ISO is linearly polarized, whereas light passing through the PC_2_ is elliptically polarized. This elliptically polarized light can be decomposed into two orthogonal polarization components of differing intensities. Due to SPM and XPM, the two polarization components acquire different nonlinear phase shifts after propagating through the fiber. The azimuth of the elliptically polarized light synthesized by PC_1_ has rotated, finally passing the PD-ISO. By adjusting PC_1_ and PC_2_, the polarization direction at the center of the light pulse is aligned with the transmission axis of PD-ISO. The pulse edge, whose polarization state is at an angle to the PD-ISO’s transmission axis, suffers significant loss. As the pulse circulates in the cavity, its duration progressively decreases, enabling the generation of ultrashort laser pulses.

In 1992, V. Matsas first constructed an Er-doped fiber laser based on NPR mode-locking, achieving self-starting mode-locking within an all-fiber structure [117]. Zhao et al. [82] incorporated NPR into a fully positive-dispersion Er-doped fiber laser for mode-locking. They obtained a square wave pulse with a repetition rate of 1.146 MHz and an output coupling ratio of 90%. The primary goals for NPR mode-locked fiber lasers are high average power, high slope efficiency, and narrow pulse widths. By removing the polarization control elements from the cavity, Xu et al. [118] reduced insertion loss in their NPR mode-locked fiber laser. The generation of the ultrashort pulse depends on twisting the fiber. The maximum output power of 134 mW was achieved at a cavity length of 26 m. Building upon Xu’s experimental work, Shang et al. [119] obtained a conventional soliton with a width of 276 fs and a pulse energy of 1.51 nJ by using a rotating polarization selective prism to alter the pulse’s polarization state. This configuration has a slope efficiency of 24.17%. The higher the pulse repetition rate, the greater the average power. Utilizing the stretched soliton effect, He et al. [120] developed an NPR mode-locked fiber laser with a repetition rate of 1.87 GHz.

Conventional NPR mode-locking in fiber lasers uses standard SMF. When elliptically polarized light propagates through the fiber, its major axis undergoes rotation due to the nonlinear Kerr effect. In order to select the required polarization state, it is essential to adjust the PC precisely. The dependence of laser stability on the polarization state indicates the necessity of controlling the external environment. Fermann et al. [124] constructed a fully polarization-maintaining NPR mode-locked Er-doped fiber laser. Their work proved that using PMF enables a stable mode-locked pulse. As shown in Figure 7b, Ye et al. [121] investigated an L-band fully polarization-maintaining fiber laser based on NPR mode-locking. Their results showed that the laser exhibited mode-locking stability with a root mean square (RMS) value of 0.27% over 12 h. The stability of fiber lasers based on NPR mode-locking can also be improved by using intelligent mode-locking solutions. As seen in Figure 7c, this solution employs intelligent algorithms to control electronic components, thereby achieving mode-locking operations [122]. Upon detecting intracavity polarization state variations, the intelligent mode-locking system responds by rapidly adjusting polarization components to counteract environmental interference. As illustrated in Figure 7d, Tong et al. [123] employed an improved particle swarm algorithm to achieve intelligent mode-locking. At a pump power of 400 mW, the pulse repetition rate reached 709.6 kHz. Intelligent mode-locking solutions have addressed the challenges with polarization adjustment in traditional NPR fiber lasers and shortened the mode-locking time. It significantly influences the development of NPR mode-locked fiber lasers.

### 4.4. Mamyshev Oscillator

**Figure 8:**
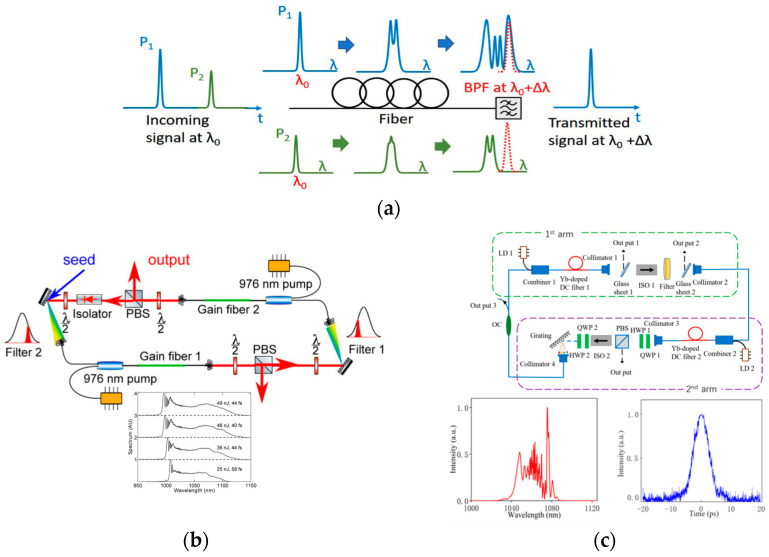
Using Mamyshev oscillator for mode-locking in fiber lasers: (**a**) schematic diagram of Mamyshev regenerator’s saturable absorption effect [126]; (**b**) a high energy Mamyshev oscillator, which is initiated [127]; (**c**) Mamyshev oscillator combined with space devices (DC fiber, doped core fiber) [128].

The high-energy pulse produced by balancing the dispersion and nonlinearity in the fiber laser cavity will still split at high pump power because of the nonlinear effect in the fiber. In response, researchers have proposed the use of Mamyshev oscillators, which can generate ultrashort pulses with peak power levels comparable to solid-state lasers. The Mamyshev regenerative amplifier was proposed by Mamyshev in 1998 [125], and then it was applied to communication field. A Mamyshev regenerative amplifier mainly includes a spectral filter and a section of nonlinear fibers, as shown in Figure 8a. When a pulsed signal, P_1_, and a noise signal, P_2_, at the same central wavelength, λ_0_, are injected into a nonlinear fiber, the higher peak power of P_1_ induces stronger SPM, resulting in greater broadening of the P_1_ pulse spectrum, reaching λ_1_ = λ_0_ + Δλ. Since the spectrum of pulse P_2_ does not extend to λ_1_, a spectral filter centered at this wavelength can be used to suppress it, thereby selectively transmitting only the energy from the spectrally broadened pulse P_1_ [126].

In 2008, Pitois et al. [129] demonstrated pulse source operation using the Mamyshev oscillator. They believed that the Mamyshev oscillator had great potential as a novel method for constructing mode-locked fiber lasers. Subsequently, Regelskis et al. [130] generated ultrashort pulses using the Mamyshev oscillator. However, it was not until 2017 that Liu et al. [127] formally defined the Mamyshev oscillator and constructed a Mamyshev oscillator with a fully normal dispersion regime. Through numerical simulations, they demonstrated that a Mamyshev oscillator constructed from standard SMF can support ultrashort pulses with peak power exceeding 10 MW. As shown in Figure 8b, they also produced an ultrashort pulse in experiments, achieving a peak power of 1 MW and a compressed pulse width of 40 fs. The Mamyshev oscillator has a 100% modulation depth. It can prevent noise and continuous light from damaging the output pulse. However, the excessively high modulation depth of the Mamyshev oscillator makes achieving self-starting mode-locking difficult. To address this issue, Wang et al. [131] achieved mode-locking by injecting seed light of a specific bandwidth into the oscillator via an external cavity. Sidorenko et al. [132] achieved self-starting mode-locking of an oscillator by introducing seed light via an external excitation arm. Additionally, as shown in Figure 8c, Han et al. [128] enabled self-starting mode-locking in the oscillator by incorporating distinct spatial devices in one arm to generate seed light through nonlinear polarization rotation.

The above reports all concern the Mamyshev oscillator’s mode-locking capabilities in the 1 μm wavelength band. Recently, researchers have applied the Mamyshev oscillator to additional wavelength bands. Olivier et al. [133] utilized a polarization-maintaining Er-doped fiber as the gain medium and obtained 93 fs pulses in the 1.5 μm band. Repgen et al. [134] demonstrated a mode-locked Er-doped Mamyshev oscillator by employing positive fiber dispersion to control the intracavity dispersion. A Mamyshev oscillator features a simple structure and high environmental robustness, making it a viable alternative to traditional titanium–sapphire lasers. Future research efforts will focus on enhancing its status as an ideal research platform by increasing its output pulse energy and peak power, and applying it in fields such as phase-stable frequency combs. Table 4 summarizes some of the achievements in fiber laser mode-locking using different SA materials.

## 5. Discussion

The SAs discussed above each possess their own advantages. The semiconductor fabrication process for SESAMs is well established. Critical SESAM properties, such as modulation depth and saturation fluence, are amenable to precise control. Owing to their semiconductor chip-packaging structure, SESAMs exhibit remarkable resistance to environmental variations. Companies like BATOP have achieved commercial-scale production of SESAMs. Furthermore, the devices can be readily integrated into fiber laser architectures. However, the recovery time of SESAMs is typically in the order of picoseconds, which limits their use in generating ultrashort pulses. The complex preparation process also limits its application range. Nanomaterials such as graphene exhibit exceptional broadband saturable absorption and ultrafast recovery times. These superior properties have facilitated numerous research achievements. This broadband saturable absorption characteristic can also be utilized for dual-wavelength synchronous mode-locking. Sotor et al. have reported a scheme for achieving synchronous mode-locking of EDFL and TDFL using graphene [141]. This will facilitate coherent synthesis in the mid infrared band to generate few cycle pulses [142]. The lack of precise and controllable nanomaterial preparation processes hinders the large-scale production of nanomaterial SAs [143]. The industrialization of graphene offers hope of improving the preparation processes of other nanomaterials. Additionally, it is necessary to investigate methods for inhibiting photodegradation, enhancing antioxidant properties, and improving hydrolysis resistance to enhance the long term stability of nanomaterial SAs [144].

When employing the NPR mode-locking scheme, switching between different pulse shapes can be achieved by adjusting the polarization controller. However, NPR technology generally requires the use of PMF to control stability. An NOLM is usually more stable than NPR. The NALM architecture readily achieves self-starting mode-locking by incorporating the gain medium within the nonlinear loop. The NOLM structure requires precise control of the loop length, which is critical for achieving the constructive interference necessary for mode-locking. The introduction of NOLM increases the cavity length, which is detrimental to generating pulses with high repetition rates and high average power. NLMMI offers advantages such as simple structure, ease of integration, and low cost. However, NLMMI cannot generate diverse pulse shapes like NPR. NLMMI mold-locking relies on precise numerical simulation and involves high design complexity. The Mamyshev oscillator achieves an exceptionally high modulation depth (100%), providing outstanding continuous wave and noise background suppression. Since Mamyshev oscillators operate in a state of high-gain saturation, the output peak power of fiber Mamyshev oscillators can reach up to the MW level. However, due to the lack of sufficient nonlinear effects in low peak power lasers to cause spectral broadening, the Mamyshev oscillator requires an initial pulse signal injection to achieve self-starting.

Emerging fields such as quantum computing and nanomanufacturing demand laser pulses with high stability, short pulse widths, and high peak power. Long wavelength lasers can be effectively absorbed by biological tissues, making them highly valuable for applications in medical fields such as ophthalmology. However, few ultrafast fiber lasers with center wavelength greater than 2 μm have been reported. The mode-locking scheme for mid-infrared fiber lasers based on SAs requires further development. In the field of communications, narrow pulse widths are essential for achieving data transmission rates exceeding hundreds of gigabits per second. Pulse width can be optimized through either intracavity pulse width control or extracavity pulse compression. Reports indicate that combining a real SA with an artificial SA can not only compress the pulse width but also enhance the output power, pulse energy, and signal-to-noise ratio of fiber lasers. To generate pulse shapes suitable for different application scenarios, it is often necessary to optimize laser cavity parameters. Artificial intelligence (AI) automatic control simplifies switching between different pulse waveforms. By integrating artificial saturable absorbers with adaptive machine learning, an automated polarization recovery system is created. It actively compensates for external disturbance-induced drift using a motorized polarization controller, thus ensuring the maintenance of mode-locking [145]. This approach promises operational performance characterized by high efficiency, stability, and precision.

## 6. Conclusions

This paper analyses passive mode-locking schemes based on various saturable absorbers. It reviews relevant research progress and current developments, including SESAMs, nanomaterials, NOLMs, NLMMI, NPR, and the Mamyshev oscillator. Their development has increased the usefulness of ultrashort pulse lasers, expanded the range of applications for ultrashort pulse fiber lasers, and greatly advanced the practical development of ultrashort fiber light sources.

Benefiting from mature preparation and characterization protocols, ultrashort pulse lasers incorporating two-dimensional nanomaterials have undergone rapid development. This progress has yielded a wealth of important research outcomes. Future advancements in mode-locked fiber lasers utilizing material SAs are anticipated across several key areas. Firstly, the operational wavelength of output pulses can be broadened, with significant potential remaining in the ultraviolet and mid-infrared spectral regions. Secondly, many nonlinear phenomena have been explored and verified. Finally, more novel nanomaterials can be synthesized and their nonlinear response characteristics can be explored.

The advancement of biological imaging technology has led to the widespread adoption of multiphoton microscopy in biological research. A high-performance ultrashort pulse laser serves as a crucial component for deep-tissue imaging. The emergence of the 1.7 μm ultrashort pulse fiber laser provides a robust foundation for further progress in multiphoton microscopy.

The field of attosecond technology is rapidly advancing, providing a direct means to probe and control electronic processes. These lasers are predominantly generated by exciting inert gases with high-intensity and short-period pulses to produce high-order harmonics. The ultrashort pulse fiber laser, notably the Mamyshev oscillator, distinguished by its ability to output high energy and short period pulses, represents a promising driving source for efficient attosecond generation.

In order to design ultrashort fiber lasers for diverse application requirements, it is essential to understand the various locking mechanisms of these SAs. This knowledge will help to advance technology towards greater maturity.

## Figures and Tables

**Table 1:** Performances of mode-locked fiber laser using an SESAM.

Configuration	Pulse Width (ps)	Pulse Energy (nJ)	Peak Power (W)	Wavelength (nm)	Pulse Profile	Repetition Rate (MHz)	Ref.
Ring	0.109	0.44	4000	1034.4	Soliton	50	[34]
Linear	0.135	4000	14,000	1582	Soliton	25.8	[37]
Ring	0.26	0.08	473	1060	SP	553	[30]
Ring	0.177	0.12	680	1034	Soliton	0.3971	[38]
Linear	1.52	2.9	1680	3434.2	Soliton	490	[39]
Ring	0.753	0.0041	5.4	1561.9	Soliton	23.5	[40]
Linear	3.6	2.4 × 10^−4^	0.062	1561	Soliton	5000	[41]
Linear	15	2.7	180	2791.3	Soliton	17.2	[42]
Linear	21	0.034	1.6	1064.1	NGP	1048	[36]
Linear	24	2.1	87.5	1034	DS	10.57	[31]
Linear	576	0.101	0.15	1061	DS	55	[43]
Linear	910	4.3	4.7	1068.76	DS	9.89	[35]

Note: Soliton: an ultrashort pulse formed when dispersion and nonlinear effects are balanced; stretched pulse (SP): a chirped pulse that has been spread for safety amplification; dissipative soliton (DS): a high-energy pulse formed when gain, loss, dispersion, and nonlinear effects are balanced; NGP: narrowband Gaussian pulse.

**Table 2:** Nanomaterial SAs for fiber laser mode-locking.

Type	CNTs	Graphene	BP	TIs	TMDs
	[44,45,46,47]	[48,49]	[50,51,52]	[53,54,55]	[56,57,58]
Atomic structure	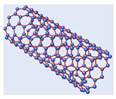	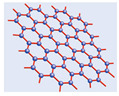	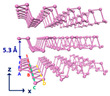	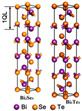	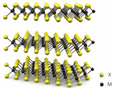
Bandgap	0.5–1 eV	0 eV	0.35–2 eV	0–0.7 eV	1–2.5 eV
Carrier lifetime	Fast: <1 ps Slow: ~250 ps	Fast: <200 fs Slow: ~1 ps	Fast: 360 fs Slow: 1.36 ps	Fast: 0.3–2 ps Slow: 3–23 ps	Fast: 1–3 ps Slow: 70–400 ps

Note: CNTs: carbon nanotubes; BP: black phosphorus; TIs: topological insulators; TMDs: transition metal dichalcogenides.

**Table 3:** Performances of mode-locked fiber laser using nanomaterials SA.

Configuration	SA	Pulse Width (ps)	Pulse Energy (nJ)	Peak Power (W)	Wavelength (nm)	Spectral Bandwidth (nm)	Ref.
Ring	SWCNT	0.226	2.35	10.4 × 10^3^	1561.8	13.9	[64]
Ring	SWCNT	0.249	0.221	83.3	1550	9.3	[100]
Linear	CNT-PVA	0.816	-	-	1562.3	3.6	[66]
Ring	CNTs	0.829	-	-	1565	3.1	[62]
Ring	CNTs	0.9	0.59	1.06 × 10^3^	1560	2.8	[63]
Ring	SWCNT	276	0.103	0.373	1064	0.57	[101]
Ring	Graphene	0.694	0.507	731	1558.35	4.21	[69]
Ring	Graphene	0.756	1.12	1.48 × 10^3^	1565	5	[68]
Ring	Graphene	2.1	0.08	38.1	1953.3	2.2	[70]
Ring	BP	0.335	0.119	173	1561	8.4	[79]
Ring	BP	0.739	0.0407	55	1910	5.8	[78]
Ring	BP	0.786	-	-	1565	3.8	[77]
Ring	BP	1.17	5.4	4.7 × 10^3^	1556.2	2.2	[74]
Linear	BP	42	25.5	610	2783	2.8	[80]
Linear	BP	-	-	-	3489	4.7	[81]
Ring	Sb_2_Te_3_	0.27	0.029	95	1561	10.3	[87]
Ring	Bi_2_Se_2_Te	0.583	29.62	5.08 × 10^3^	1561.5	3.6	[89]
Ring	Bi_2_Se_3_	0.63	0.211	334.9	1565	7.9	[88]
Ring	Bi_2_Te_3_	1.21	-	-	1158.4	2.69	[86]
Ring	Bi_2_Te_3_	1.26	-	-	1909.5	5.64	[83]
Linear	Bi_2_Se_3_	46	0.756	16.4 × 10^3^	1031.7	2.5	[75]
Ring	WS_2_	0.66	-	-	1557	4.0	[93]
Ring	VSe_2_	0.67	0.441	620	1561.5	4.1	[76]
Ring	WSe_2_	0.698	0.21	300.6	1555.2	4.6	[95]
Ring	PdS_2_	0.766	0.045	58.5	1566	4.16	[96]
Linear	MoS_2_-PVA	0.96	0.065	67.7	1535–1565	3.0	[92]
Linear	MoSe_2_	1	0.123	12.3	1558.35	2.9	[94]
Linear	MoS_2_	800	1.41	1.76	1054.3	2.7	[91]

Note: CNT-PVA: CNT-Polyvinyl Alcohol; MoS_2_-PVA: MoS_2_-Polyvinyl Alcohol.

**Table 4:** Performances of mode-locked fiber laser using SA materials.

Configuration	SA	Pulse Width (ps)	Pulse Energy (nJ)	Peak Power (W)	Wavelength (nm)	Repetition Rate (MHz)	Ref.
Ring	NALM	0.215	0.214	1 × 10^3^	1030	700.1	[104]
Ring	NALM	0.344	0.3	870	1027	10	[105]
Ring	NALM	0.615	32	52 × 10^3^	1030	2.47	[106]
Ring	NOLM	1.39	0.718	51.6 × 10^5^	1559.7	0.8	[135]
Ring	NOLM	2.8	0.0838	29.9	2017.33	1.51	[136]
Ring	NALM	57.3	2.85	23	717.5	14.58	[109]
Ring	NLMMI	1.8	0.0305	19.1	1559	49.8	[111]
Linear	NLMMI	2.3–2.5	-	-	1529/1558	5.66	[115]
Ring	NLMMI	46	0.0368	0.8	1958	8.58	[114]
Ring	NLMMI	136	-	-	1562	40	[137]
Linear	NLMMI + NPR	-	-	-	1064	12.8	[112]
Ring	NPR	0.037	0.31	8.29 × 10^3^	1550	225	[138]
Linear	NPR	0.117	0.06	513	1550	12.1	[120]
Ring	NPR	0.276	1.51	5.16 × 10^3^	1584.8	32.5	[119]
Ring	NPR	0.89	0.75	840	1950	248	[139]
Linear	NPR	1.2	0.11	91.7	1573	115	[140]
Ring	NPR	1.25	0.0262	100	1584.2	3.9	[121]
Ring	Mamyshev	0.056	83.5	1.15 × 10^6^	1022.2	9.43	[131]
Ring	Mamyshev	0.093	31.3	60 × 10^3^	1550	7.35	[133]
Linear	Mamyshev	0.208	3.55	17.1 × 10^3^	1965	15.027	[134]
Linear	Mamyshev	3.1	0.73	235	1060	14.52	[130]
Linear	Mamyshev	4.16	8.6	2.07 × 10^3^	1062.53	15.18	[128]
Ring	Mamyshev	6	50	1 × 10^6^	1035	17	[127]

Note: NALM: nonlinear optical amplifier loop mirror; NOLM: nonlinear optical loop mirror; NLMMI: nonlinear multimode interference; NPR: nonlinear polarization rotation. (A composite SA that incorporates an artificial SA can still be classified as an artificial SA. The “NLMMI + NPR” configuration is a case in point).

## Data Availability

Not applicable.
